# Clinical characteristics, diagnosis, and outcomes of orbital biopsies in a single Irish centre

**DOI:** 10.1007/s11845-022-03133-4

**Published:** 2022-08-22

**Authors:** Siân Kneafsey, Terence MacSwiney, Clare McCloskey, Conor O’Keane, Tim Fulcher

**Affiliations:** 1 Ophthalmology Department, Mater Misericordiae University Hospital, Dublin, Ireland; 2 Histopathology Department, Mater Misericordiae University Hospital, Dublin, Ireland

**Keywords:** Biopsy, Diagnosis, Histopathology, Orbital, Preoperative, Postoperative

## Abstract

**Aims:**

To review the distribution of histopathological diagnoses and visual outcome of orbital biopsy in an Irish tertiary referral centre over a 10-year period.

**Methods:**

This was a retrospective, clinical-histopathological case series. Clinical records of all patients who underwent orbital biopsy between January 2008 and January 2018 in the Mater Misericordiae University Hospital were reviewed using data collected from theatre logbooks and hospital-based medical records.

**Results:**

A total of 83 orbital biopsies in 77 patients were included for analysis in this study. The mean age was 55.7 ± 18.41 years. The mean follow-up period was 1.87 ± 2.097 years. The most common presenting symptoms and signs were pain (22.3%) and proptosis (27.6%). Most lesions were located in the extraconal space (65%), with incisional biopsy (65%) being the most common technique used to gain a sample for histopathological diagnosis. Histopathology analysis of the biopsies revealed malignant tumours (27, 32.5%), benign tumours (7, 8.4%), inflammation (26, 31.3%), and other diagnoses (23, 27%). Excluding patients who underwent exenteration procedures, no study patients suffered visual loss following orbital biopsy.

**Conclusions:**

Orbital biopsy serves as a safe diagnostic tool in managing orbital diseases. The breakdown of diagnosis in our patients is in line with international studies. No patients in our series suffered vision loss as a result of their orbital biopsy. This emphasises its use as a safe procedure in the diagnosis and management of patients with the orbital disease. Our data provides helpful guidance to clinicians when counselling patients for orbital biopsy.

## Introduction

Orbital space-occupying lesions in adults comprise a broad spectrum of benign and malignant entities. The differential diagnosis of an orbital mass can a pose challenging question to the investigating clinician. Symptoms and signs can be non-specific. The lesion may not be visualised and may not be amenable to external physical examination. The management of orbital lesions is further complicated by a small but clinically significant proportion of malignant masses. These lesions can be both sight and life-threatening [1]. The delayed diagnosis of some aggressive malignancies, which masquerade clinically as inflammatory lesions, can lead to delayed management and and ineffective treatment. The orbital biopsy is an important diagnostic test to diagnose orbital space-occupying lesions [2] accurately. Histopathological diagnosis can be used to correlate clinical and radiological suspicion. Clinical history, examination, radiological imaging, and histopathological analysis can be used to determine a diagnosis and create an appropriate patient-specific management plan.

The literature has a wide variance in the incidence of space-occupying lesions depending on the source of the material reviewed [3]. Some studies in the past century have only included pathology reviews of orbital biopsy specimens [4–7], while some only reviewed cross-sectional radiological imaging [8]. More recently, several centres have analysed their single centre experience [3, 9–14], combining clinical, radiological findings, and histopathological results. Despite this, limited data remains to discuss the Irish experience of orbital biopsy, clinical and histopathological diagnoses and postoperative visual outcomes.

Our article aims to describe the distribution of histopathological diagnoses of biopsied orbital lesions and visual outcomes in a tertiary referral centre over a 10-year period and compare the associated clinical characteristics of the lesions that may help the clinician in the differential diagnosis.

## Methods

This was a retrospective, single-centre clinical-histopathological case series at the Mater Misericordiae University Hospital (MMUH), Dublin, Ireland. All patients undergoing orbital biopsy between January 2008 and January 2018 were enrolled. These cases were identified from theatre logbooks and hospital inpatient enquiry (HIPE). Data was taken from the hospital-based electronic patient record and the physical medical charts. The study received the approval of the hospital ethics committee. The tenets of the Helsinki agreement were followed throughout.

### Preoperative examination

All patients underwent a complete eye examination preoperatively. Demographic factors, presenting symptoms, and signs were recorded. The ocular data included laterality (bilateral and unilateral), affected eye (right eye and left eye), and best-corrected Snellen visual acuity. The tumour data included orbit location (intraconal, extraconal, and diffuse) and anteroposterior orbital location (anterior, middle, and posterior). Any medical comorbidities, previous surgery, and all medications were recorded.

### Inclusion criteria

Inclusion criteria in our study included age > 16 years and any patient undergoing a clinically indicated orbital biopsy between our dates.

### Postoperative examinations

Histopathological results, visual outcome, and time to last post-biopsy follow-up were collected. The final diagnosis was determined by history, ocular findings, diagnostic imaging, and histopathologic analysis.

The primary diagnosis in each case was categorised into one of several major groups of lesions (malignant, benign, inflammation, infectious, and others) as in previously published large cohort studies by Shields et al. [3]. The tumour management was assessed and recorded (incisional biopsy and excisional biopsy). Follow-up data, including the date of the last review, were collected.

## Results

A total of 83 orbital biopsies in 77 patients were included for analysis in this study. Table 1 displays the demographics of our study cohort. The mean age was 55.7 ± 18.41 years (a range of 16.2–96.0 years old). Nine patients (10.8%) in our study had a predisposing orbital condition or history of orbital surgery prior to their presenting orbital mass. The mean follow-up period was 1.87 ± 2.097 years.

**Table 1:** Demographic factors and clinical details of all orbital biopsies

***Total n = 83*** ***(n %)***	
**Age**	55.7
***Gender***	
Male	49 (59)
Female	34 (40.9)
*Location of lesion*	
Intraconal	23 (27.7)
Extraconal	54 (65)
Diffuse	6 (7.22)
*Biopsy*	
Incisional	54 (65)
Excisional	29 (34.9)

In our study, 17 patients (19.7%) had a known underlying diagnosis of malignancy at the time of presentation. These included lymphoma (5, 5.8%), breast cancer (5, 5.8), malignant melanoma (2, 2.3%), meningioma (2, 2.3%), multiple myeloma (1.1%), leukaemia (1, 1.1%), and renal cancer (1, 1.1).

**Fig. 1:**
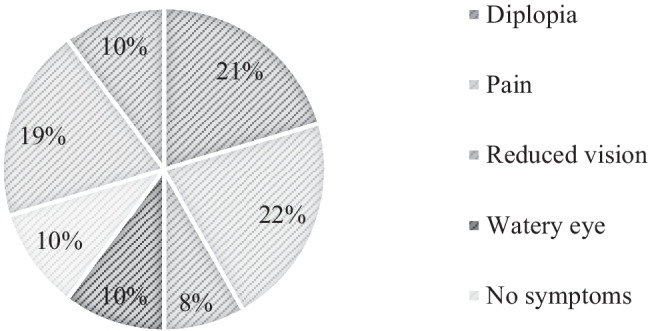
Breakdown of presenting symptoms of all orbital biopsies

**Fig. 2:**
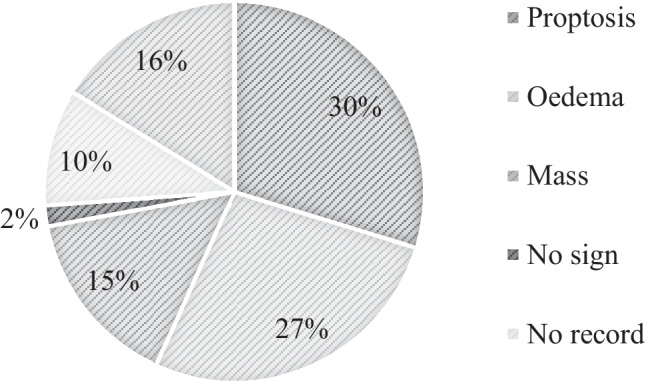
Breakdown of presenting signs of all orbital biopsies

Figures 1 and 2 summarise our patient cohort’s most common presenting symptoms and signs. Patients often presented with more than one symptom or sign; pain (22.3%) and proptosis (27.6%) were the most common presenting symptoms and signs, respectively. A total of 10% [9] of patients had no orbital symptoms at the time of biopsy. A total of 2% [2] of patients had no signs on examination at the time of biopsy.

Table 1 displays demographic factors and clinical details of all orbital biopsies. The majority of lesions were located in the extraconal space (65%), with incisional biopsy (65%) the most common technique used to gain a sample for histopathological diagnosis.

Table 2 demonstrates the variety of histopathological diagnoses of all orbital biopsies in our study. Malignant lesions (27, 32.5%) and inflammatory orbital diseases (26, 31.3%) were the most common group of histopathological diagnoses. Specifically, nonspecific orbital inflammatory disease (15, 18.1%), lymphoproliferative disease (9, 10.1%), and haemangioma (8, 8.9%) were the four most common diagnoses. Regarding the lymphoproliferative lesions, there were eight diffuse B cell/non-Hodgkin lymphoma and one follicular lymphoma CD20 + .

**Table 2:** Breakdown of histopathological diagnoses of all orbital biopsies

***Category***	***Diagnosis***	***Total n = 83*** ***(n %)***
Malignant tumours	Lymphoproliferative disorder	9 (10.8)
	Metastases	5 (6)
	Squamous cell carcinoma	5 (6)
	Basal cell carcinoma	4 (4.8)
	Sarcoma	1 (1.2)
	Malignant melanoma	1 (1.2)
	Merkle cell carcinoma	1 (1.2)
	Neuroendocrine carcinoma	1 (1.2)
	*Total*	*27 (32.5%)*
Benign tumours	Haemangioma (benign, cavernous)	8 (9.6)
	Neurofibroma	3 (3.6)
	Meningioma	2 (2.4)
	Schwannoma	1 (1.2)
	Reactive lymphoid process	1 (1.2)
	Eosinophilic granuloma	1 (1.2)
	*Total*	*7 (8.4%)*
Inflammation	Nonspecific orbital inflammatory disease	15 (18.1)
	Thyroid eye disease	5 (6)
	Granulomatosis with polyangiitis	2 (2.4)
	Sjogren’s syndrome	2 (2.4)
	Eosinophilic polyangiitis	1 (1.2)
	Foreign body giant cell reaction	1 (1.2)
	*Total*	*26 (31.3%)*
Other	Dermoid cyst	2 (2.4)
	Lacrimal cyst	2 (2.4)
	Normal	2 (2.4)
	Dacryoadenitis	2 (2.4)
	Benign conjunctival cyst	1 (1.2)
	Inclusion cyst	1 (1.2)
	Cyst of Moll	1 (1.2)
	Latent TB	1 (1.2)
	Fibrotic changes	1 (1.2)
	Varices	1 (1.2)
	*Total*	*23 (27%)*

Within our study, there were 11 lacrimal gland biopsies taken. Results included lymphoma (3, 27.2%**)**, Sjogren’s syndrome (2, 18.1%), inflammatory diseases (2, 18.1%), sarcoma (1, 9%), eosinophilic polyangiitis (1, 9%), inclusion cyst (1, 9%), and dacryoadenitis (1, 9%).

Mean preoperative best-corrected visual acuity was 0.21 ± 0.24. Mean postoperative best-corrected visual acuity was 0.21 ± 0.24. There were a total of eight exenterations included in our study. Excluding patients who underwent exenteration procedures, no study patients suffered visual loss following orbital biopsy.

## Discussion

This study represents the most extensive case series to our knowledge, specifically reporting the demographic factors, histopathological diagnoses, and visual outcome of orbital biopsies in Ireland to date.

### Visual outcome

Visual outcome serves as one of the primary measures in assessing the safety of orbital biopsy. While visual loss is a devastating complication after orbital surgery, it represents less than 1% of all orbital surgery performed [14]. Visual outcomes following orbital biopsy are not widely reported; however, while complications include reduced vision, significant visual loss is considered very rare [15, 16]. When explicitly considering orbital biopsy procedures, the rate of postoperative blindness was 0–0.87% [14, 15]. Our study did not observe any visual loss following orbital biopsy. We acknowledge that there are many other components to visual function, including visual field, contrast and colour, and visual acuity alone does not encompass visual function. While we did not record specific visual checks in the immediate post-op period, we did record the visual acuity of each patient during their follow-up appointment. We found no patient to have suffered any new visual loss, suggesting that orbital biopsy is a safe diagnostic procedure in managing patients with the orbital disease.

### Orbital disease

A large variation of orbital diseases has been reported in the literature. [1, 3, 7, 9, 10, 12, 15, 16]. Ting et al. [9], Shields et al. [3], and Jamison et al. [15] all reported nonspecific orbital inflammation and lymphoproliferative disease as the two most common orbital diseases. Similarly, our study found nonspecific orbital inflammatory diseases (15, 15.6%) and lymphoproliferative disease (9, 9.4%) the most common orbital diseases reported during our 10-year study period.

### Nonspecific inflammatory disease

Nonspecific orbital inflammatory disease is a benign, non-infectious, non-neoplastic, space-occupying, inflammatory condition of the orbit and peri-orbit without identifiable local or systemic causes. The exact aetiology of this inflammatory disorder is unknown [17]; however, these conditions tend to respond clinically to systemic steroid treatment. It has been suggested that many factors may be involved, including genetic, viral, autoimmune, and environmental triggers [2, 17–19].

Mombaerts et al. [2], Rootman [11], and Fujii et al. [20] have all attempted to create different classification systems for nonspecific orbital inflammatory disease. However, given these lesions' inconsistent clinical, pathological, and imaging characteristics, none of these systems is used in general clinical practice.

The most common clinical features of nonspecific inflammatory disorders include orbital swelling, proptosis, pain, and restriction of eye movements. These are the most common presenting orbital symptoms and signs in adults [17, 21]. In our study, proptosis (8, 53.3%), swelling (7, 46.6%), diplopia (5, 30%), and restriction of eye movements (3, 20%) were the most common presenting symptoms and signs of nonspecific inflammatory disorders.

In terms of gender and age demographics, some older studies suggested a female preponderance [18]; however, a recent 2019 study by Eshraghi et al. [17] found males and females to be nearly equal. Our study found a male predominance of orbital inflammatory diseases (66%, 10) and a mean age of 44 years.

### Specific inflammatory disease

Regarding specific inflammatory diseases, we wish to highlight two biopsies in our case series. In the case of the foreign body granulomatous reaction, this patient presented with multiple episodes of severe pain, proptosis, and restricted eye movements due to orbital inflammation. This patient had undergone several biopsies in other sites prior to his referral to our centre. Analysis of the histopathology revealed foreign body reactions. Despite multiple methods of imaging, a foreign body could not be seen. The patient eventually underwent an exenteration elsewhere due to intractable pain and a fragment of the pencil was found.

In the case of latent tuberculosis, the patient in question had no prior diagnosis of TB. He had not been on any regular medications prior to his presentation. Histopathological analysis of the biopsy specimen revealed an inflammatory pseudotumour and a diagnosis of Latent TB was made. The patient underwent treatment with methylprednisolone, prednisolone, and isoniazid for 9 months.

### Incidental findings

The majority of patients who underwent orbital biopsy within our study period presented with symptoms including pain, diplopia, and reduced vision. Interestingly, a proportion of patients (10%, 9) described no symptoms at the time of biopsy.

Analysis showed these nine patients presented with signs including mass lesions without obstruction or visual changes (6, 6.9%), proptosis (2, 2.3%), and ptosis (1, 1.1%). Diagnoses of these patients include haemangioma [3], SCC [2], Merkel cell carcinoma [1], atypical lymphoid hyperplasia [1], dermoid cyst [1], and neurofibroma [1].

### Malignancy and metastatic disease

The diagnosis of orbital malignancy and ocular metastases is a challenging task. In our study, 17 patients (19.7%) had a known underlying diagnosis of malignancy at the time of presentation. These included lymphoma (5, 5.8%), breast cancer (5, 5.8), malignant melanoma (2, 2.3%), meningioma (2, 2.3%), multiple myeloma (1.1%), leukaemia (1, 1.1%), and renal cancer (1, 1.1%). Our study found seven patients with metastatic ocular tumours (five with breast metastases, one with multiple myeloma, and one with renal metastases). These findings are in keeping with the most common primary cancer site reported across the literature as breast carcinoma [3, 22, 23].

One study by Eldesouky and Elbakary [22] of orbital metastatic disease found that nearly a quarter of patients had no prior cancer diagnoses, with similar figures being reported by Goldberg et al. [23] and Garrity et al. [24]. Interestingly, in our study four patients had no previous diagnosis at the time of biopsy. This further highlights the importance of undertaking orbital biopsy as part of the diagnostic workup. Of these cases, three patients were diagnosed with non-Hodgkin’s lymphoma, with one patient subsequently diagnosed with metastatic lobular carcinoma of a breast primary.

Rootman [11] and Goldberg et al. [23] both classified the features of orbital metastasis into four categories: infiltrative, including restriction of eye movements or enophthalmos; mass effect, including proptosis or globe displacement; inflammatory, including pain and erythema; and functional, where the cranial nerve findings are out of proportion to the degree of orbital involvement. They found that infiltrative presentations were the most common. We found that diplopia (3, 43%) and proptosis (2, 29%) were the most common features. As discussed earlier, these are nonspecific and have the same presenting symptoms and signs as nonspecific inflammatory diseases and benign lesions. This further supports the evidence that th*e* definitive diagnosis of an orbital metastasis requires tissue diagnosis and is of diagnostic benefit to all patients with an orbital mass.

### Normal histology

Histopathological analysis revealed normal histology for two biopsies. One patient, referred from another centre for biopsy, had presented with decreased vision in both eyes and systemic symptoms of fatigue, night sweats, and myalgia. No definitive diagnosis was established from the orbital biopsy.

The second patient had a nerve sheath biopsy following presentation with loss of vision. Examination revealed bilateral optic neuropathy with disc swelling. A nerve sheath biopsy revealed normal histology and did not give an explanation for his signs and symptoms.

As demonstrated in this study, orbital biopsy serves as a valuable and safe diagnostic procedure in managing patients with the orbital disease. In some cases, the histology of orbital biopsy can be normal. This further emphasises the importance of performing a biopsy in doubtful cases as it provides reassurance to the patient and helps to obviate the need for unnecessary treatment in case of misdiagnoses.

### Repeat biopsies

There were six patients in our study who underwent a repeat biopsy within our study period. Three of these patients underwent repeat biopsy due to poor response to treatment in an inflammatory condition. In these cases, a repeat biopsy was indicated to reconfirm the initial diagnosis and ensure there was not a missed diagnosis of a malignant condition. All three showed the same histopathological diagnosis on repeat biopsy. Two patients underwent repeat biopsy due to recurrence of disease, specifically lymphoma and metastatic breast cancer. One patient underwent a repeat biopsy to ensure wider excision of diseased tissue.

## Limitations

Long-term follow-up remained unavailable for a cohort of patients due to the tertiary referral service setting of the Mater Misericordiae University Hospital.

Although the Mater Misericordiae University Hospital serves as the tertiary referral centre for orbital diseases in the North Leinster catchment area, we acknowledge that our study did not capture all the orbital biopsies in the North Leinster region as some straightforward cases may have been managed independently in other local ophthalmic units. Nonetheless, we believe that our study captured the majority of cases to provide a good overview of the histopathological diagnosis, visual outcome, and safety of orbital biopsy in the North Leinster area.

## Conclusion

Orbital biopsy serves as a safe diagnostic tool in managing orbital diseases. The breakdown of diagnosis in our patients is in line with international studies. No patients in our series suffered vision loss as a result of their orbital biopsy. This emphases its use as a safe procedure in the diagnosis and management of patients with of orbital disease. Our data provides helpful guidance to clinicians when counselling patients for orbital biopsy.
